# Effect of Annealing in Air on the Structural and Optical Properties and Efficiency Improvement of TiO_2_/Cu_x_O Solar Cells Obtained via Direct-Current Reactive Magnetron Sputtering

**DOI:** 10.3390/ma18040888

**Published:** 2025-02-18

**Authors:** Grzegorz Wisz, Maciej Sibiński, Mirosław Łabuz, Piotr Potera, Dariusz Płoch, Mariusz Bester, Rostyslav Yavorskyi

**Affiliations:** 1 Institute of Materials Engineering, University of Rzeszow, Pigonia 1, 35-310 Rzeszow, Poland; gwisz@ur.edu.pl (G.W.); ppotera@ur.edu.pl (P.P.); dploch@ur.edu.pl (D.P.); 2 Department of Material and Environmental Technology, Tallinn University of Technology, Ehitajate tee 5, 19086 Tallinn, Estonia; maciej.sibinski@p.lodz.pl; 3 Department of Semiconductor and Optoelectronic Devices, Łódź University of Technology, Al. Politechniki 10, 93-590 Łódź, Poland; 4 Institute of Physics, University of Rzeszow, Pigonia 1, 35-310 Rzeszow, Poland; mbester@ur.edu.pl; 5 Department of Physics and Chemistry of Solid State, Vasyl Stefanyk Precarpathian National University, T. Shevchenko Str. 57, 76-018 Ivano-Frankivsk, Ukraine; roctyslaw@gmail.com

**Keywords:** TiO_2_/Cu_x_O solar cells, thin films, post-annealing treatment, efficiency improvement

## Abstract

In this study, four various titanium dioxide/cuprum oxide (TiO_2_/Cu_x_O) photovoltaic structures deposited on glass/indium tin oxide (ITO) substrates using the direct-current (DC) reactive magnetron sputtering technique were annealed in air. In our previous work, the deposition parameters for different buffer layer configurations were first optimized to enhance cell fabrication efficiency. In this paper, the effects of post-deposition annealing at 150 °C in air on the optical properties and I-V characteristics of the prepared structures were examined. As a result, significant changes in optical properties and a meaningful improvement in performance in comparison to unannealed cells were observed. Air annealing led to an increase in the reflection coefficient of the TiO_2_ layer for three out of four structures. A similar increase in the reflection of the Cu_x_O layer occurred after heating for two out of four structures. Transmission of the TiO_2_/Cu_x_O photovoltaic structures also increased after heating for three out of four samples. For two structures, changes in both transmission and reflection resulted in higher absorption. Moreover, annealing the as-deposited structures resulted in a maximum relative increase in open-circuit voltage (V_oc_) by 294% and an increase in short-circuit current (I_sc_) by 1200%. The presented article gives some in-depth analysis of these reported changes in character and origin.

## 1. Introduction

Advancements in technology and enhancements in energy efficiency can significantly reduce CO_2_ emissions, helping to mitigate the climate crisis. Recently, there has been increasing interest in CuxO (Cu_2_O—cuprous oxide; CuO—cupric oxide) and TiO_2_ (titanium oxide) films, which are fabricated in various configurations for solar cell applications. These include perovskites [1], PEC (photoelectrochemical) cells for unassisted solar water splitting [2], DSSCs (dye-sensitized solar cells) [3], and CZTS (Cu_2_ZnSnS_4_) cells [4]. Among the various convenient methods for thin film deposition, many researchers use magnetron sputtering (MS) with different parameters for diverse applications of Cu_x_O [5,6] and TiO_2_ layers [7,8]. Furthermore, many authors, including our group, investigate TiO_2_/Cu_x_O heterostructures due to their complementary properties as promising materials for photovoltaic devices [9,10,11,12,13,14]. Since TiO_2_ has a band gap energy (E_g_) of 2.96–3.2 eV [15,16,17], it only absorbs ultraviolet light, which accounts for approximately 7% of the solar spectrum. Therefore, it is advantageous to use direct band gap Cu_x_O-based layers, which can absorb a larger portion of the sunlight spectrum. The Eg values for CuO range from 1.0 to 2.6 eV, while for Cu_2_O, they range from 1.8 to 2.2 eV [18,19,20,21,22]. The magnetron sputtering technique enables the deposition of TiO_2_- and Cu-based thin films under controlled conditions. Various process variants have been explored for the deposition of TiO_2_/Cu_x_O thin films, including co-sputtering of TiO_2_ and Cu targets in a reactive atmosphere or sequential sputtering of TiO_2_ followed by Cu deposition. Both approaches provide flexibility in tuning film composition, thickness, and morphology, allowing for the optimization of device performance. Annealing treatments are crucial for enhancing the performance of TiO_2_/Cu_x_O solar cells by influencing their structural, optical, and electrical properties [23,24].

The deposition of a semiconductor thin film on a substrate introduces numerous defects due to various factors, including the purity of the substrate and the deposited material, the vacuum level in the sputtering process, the purity of the gases used, the deposition rate, the temperature of the substrate, etc. [25,26]. Defects may arise from the formation of a non-equilibrium state in the film, which can be reduced through moderate heat treatment. While annealing generally decreases the non-equilibrium state and lowers the film’s specific resistance, annealing in air at temperatures above 200 °C may have the opposite effect due to oxidation at the surface of the islands that form the film.

In this paper, the authors analyzed the impact of annealing in air changes in the optical and photovoltaic properties of TiO_2_/Cu_x_O cells with Ti and Ti/Cu buffer layers in various configurations. As a result, a significant change in parameters was observed after annealing samples at 150 °C for one hour, despite the cells being originally deposited on a substrate at 300 °C. Annealing in the air has proven to be a technologically simple and cost-effective method for significantly enhancing the efficiency of as-deposited TiO_2_/Cu_x_O photovoltaic structures being investigated. In this paper, the authors showed, for the first time, the influence of annealing on the efficiency of TiO_2_/Cu_x_O photovoltaic structures with different buffer layer configurations.

## 2. Materials and Methods

In this paper, we present the results of annealing four selected TiO_2_/Cu_x_O thin film solar cells for which the preparation methods were described in detail in our previous work [27]. Annealed TiO_2_/Cu_x_O cells were grown by reactive direct-current magnetron sputtering (DC-MS). For all samples, the substrate temperature during the deposition process was consistently maintained at 300 °C. An indium tin oxide (ITO)-coated glass substrate with a surface resistance of 13–15 Ω/sq as well as n-type silicon wafers with a <100> crystal orientation was used. Titanium (99.995% purity) and copper (99.99% purity) targets, procured from Testbourne B.V. (Helmond, The Netherlands), were used in the deposition process. Identical process parameters were applied for the deposition of both TiO_2_ and Cu_x_O layers across all samples. Samples being investigated differed in the type and the location of the buffer layers, as follows: sample #1—Cu buffer layer located between TiO_2_ and Cu_x_O layers; sample #2—Ti buffer layer located between TiO_2_ and Cu_x_O layers; sample #3—Ti buffer layer located between ITO and TiO_2_ layers, and additionally, Cu buffer located between TiO_2_ and Cu_x_O layers; and sample #4—Ti buffer located between ITO and TiO_2_ layers, and additionally, Ti-Cu buffer located between TiO_2_ and Cu_x_O layers [27]. To investigate the effect of annealing, all samples were heated in air at 150 °C for 1 h using a Nabertherm LH04 furnace (Lilienthal, Germany) with automatic temperature control. The morphology and cross-sectional SEM images of TiO_2_/Cu_x_O structures were obtained using a Helios NanoLab 650 scanning electron microscope from FEI (FEI Company, Hillsboro, OR, USA; Thermo Fisher Scientific Inc., Waltham, MA, USA). Surface quality was carried out using the software apparatus Gwyddion v 2.48. The spectra of transmission and reflection for structures were obtained with the use of the Cary 5000 spectrometer in the range of 180–2500 nm. The I-V characteristics of all solar cells were measured using a Keithley 2602 I-V meter in normal operating conditions (NOCTs).

## 3. Results

### 3.1. Structural Characterization

**Figure 1:**
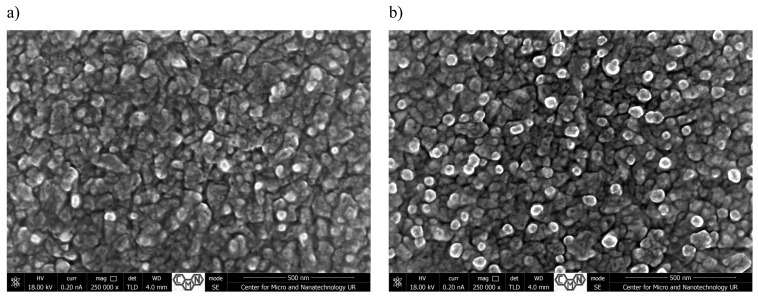

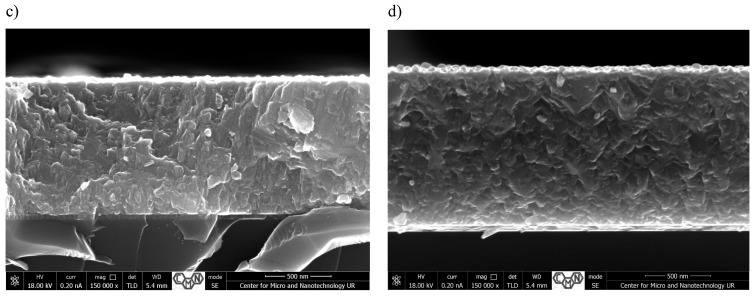
Surface morphology (**a**,**b**) and cross-sectional SEM images (**c**,**d**) of TiO_2_/Cu_x_O solar cells before (**a**,**c**) and after annealing (**b**,**d**) for sample #1.

**Figure 2:**
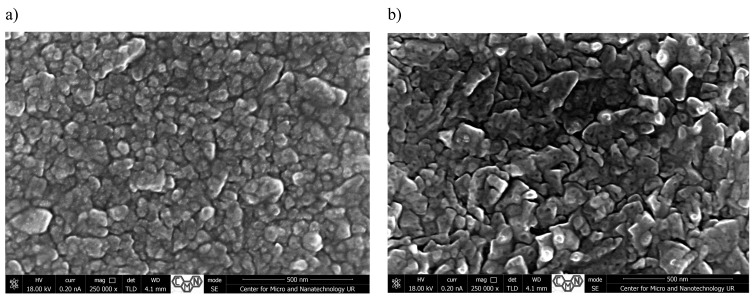

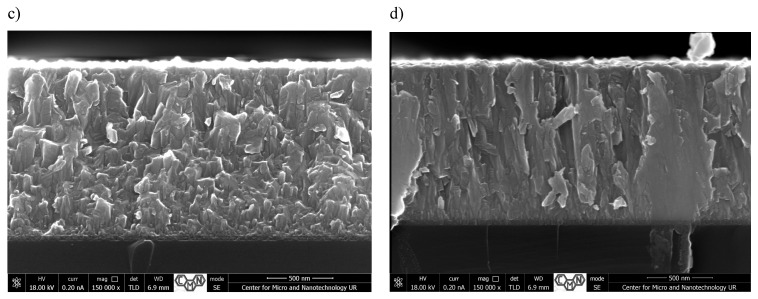
Surface morphology (**a**,**b**) and cross-sectional SEM images (**c**,**d**) of TiO_2_/Cu_x_O solar cells before (**a**,**c**) and after annealing (**b**,**d**) for sample #2.

**Figure 3:**
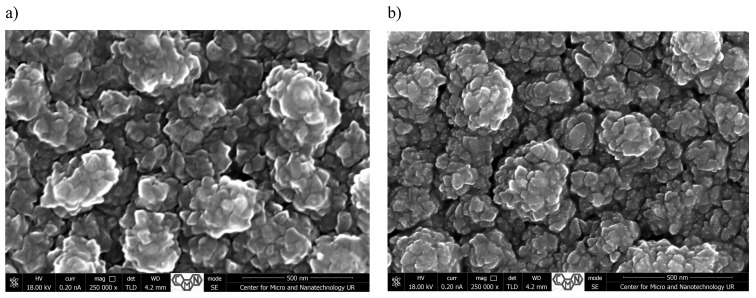

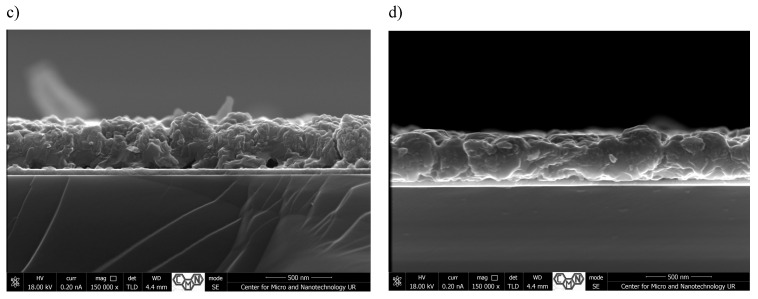
Surface morphology (**a**,**b**) and cross-sectional SEM images (**c**,**d**) of TiO_2_/Cu_x_O solar cells before (**a**,**c**) and after annealing (**b**,**d**) for sample #3.

**Figure 4:**
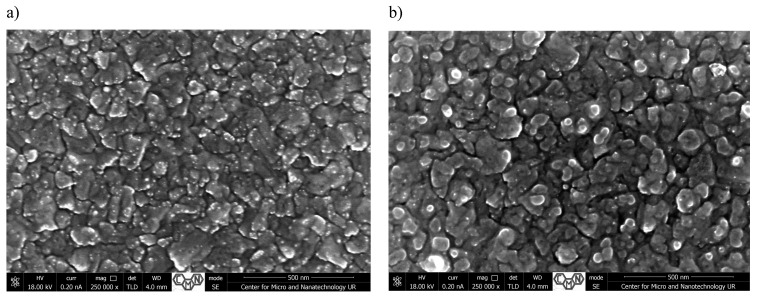

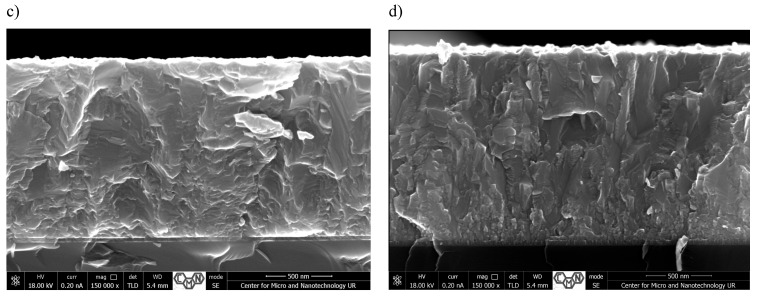
Surface morphology (**a**,**b**) and cross-sectional SEM images (**c**,**d**) of TiO_2_/Cu_x_O solar cells before (**a**,**c**) and after annealing (**b**,**d**) for sample #4.

Figure 1, Figure 2, Figure 3 and Figure 4 present SEM images of thin films of TiO_2_/Cu_x_O solar cells before (a,c) and after annealing (b,d). The morphology and cross-sectional SEM images of as-deposited (_AD) and heated in air (_H) TiO_2_/Cu_x_O structures were obtained using a Helios NanoLab 650 scanning electron microscope from FEI. The mathematical analysis of the film’s surface quality was carried out using the software apparatus Gwyddion v 2.48 (Table 1).

The interaction of atmospheric oxygen with the structure surface during annealing at 150 °C for 1 h also contributes to the morphological changes observed in all samples, as shown in Figure 1, Figure 2, Figure 3 and Figure 4. The nature of oxidation processes depends both on the degree of perfection of the crystal structure and the orientation of the crystallites. Thus, if unheated, TiO_2_/Cu_x_O films (sample #1) deposited on a silicon substrate have a more uniform surface with surface grains of 40–75 nm in size (Figure 1a). In SEM analysis, a root mean square (R_q_) is the most widely used parameter to characterize surface roughness. The average roughness (R_a_) of 130.3 nm and a root mean square (R_q_) of 177.6 nm were observed for sample #1. Then, annealing at the temperature of T = 150 °C leads to the formation of small, rounded islands with slightly smaller sizes (30–60 nm) while increasing the surface roughness (Table 1). Such an increase may be associated with the presence of an oxide phase in defective places—microcracks, pores, and increased impurity concentration (Figure 1b). In contrast to the surface of the thin film, changes were also observed in cross-sections (Figure 1c,d).

For sample #2 (Figure 2), the same situation can be observed as for sample #1. The surface of these two samples is somewhat similar but differs in the form of surface nano-objects. For sample #1 (Figure 1), the spherical formation of objects is more inherent; in turn, for sample #2, the shape of objects is more reminiscent of differently oriented pyramids. After annealing, there is a decrease in the size of the surface nano-objects, which, before annealing, were in the range of 80–135 nm, as can be seen in Figure 3a, and in the range of 70–120 nm after annealing (Figure 2b). There is also a change in the average roughness of the film, which is 126.1 nm before annealing and 149.1 after annealing. The root mean square also increases from 172.7 nm to 200.6 nm. Then, being centers of oxidation, the islands of the new phase grow rapidly, completely covering almost the entire surface of the film (Figure 2b). In contrast to polycrystalline films, the thermal annealing of epitaxial films in air leads to the formation of an oxide phase, the “focuses” of which at the initial stages appear not in the intergranular boundaries, as it would seem, but in separate places on the microcrystallites [28]. As the temperature or annealing time increases, individual phases grow and cover the entire surface of the main matrix.

In Figure 3 (sample #3), a spherical formation on the surface of the film can be seen. Such spheres were formed into separate conglomerates of various sizes from 200 to 250 nm. In the heterosurface system, if there is a constant lattice mismatch between the implanted material and the substrate, initial growth can occur layer by layer. However, a thicker layer has a greater value of elastic energy, and there is a tendency to decrease it by the formation of isolated islands. During further growth, coalescence occurs, in which larger islands grow due to diffusional redistribution of material, which leads to the reduction in and disappearance of small islands and the formation of conglomerates [29]. The consequence of such a process is the formation of conglomerates of such volume, in which the formation of dislocations of unconformity is energetically beneficial.

After annealing, the size of the conglomerates does not change significantly, but more distinct faces of spherical formations appear (Figure 3b). The average roughness of sample #3 before annealing is 171.9 nm, and it is 145.6 nm after annealing, while the root mean square is 222.5 nm and 193.2 nm, respectively.

The surface of sample #4 is very similar to sample #2 and represents pyramids of different orientations (Figure 4). The size of such formations varies from 20 to 125 nm and does not change after annealing. The average roughness of the sample before annealing (Figure 4a) is 135.0 nm and the root mean square roughness is 185.6 nm; after annealing, these parameters do not change significantly and are 133.6 nm and 186 nm, respectively (Figure 4b). Generally, in the case of all samples, we observed a decrease in the number of structural defects, mainly within the CuxO layer, which is consistent with the results obtained by the authors in [23].

### 3.2. Optical Characterization

**Figure 5:**
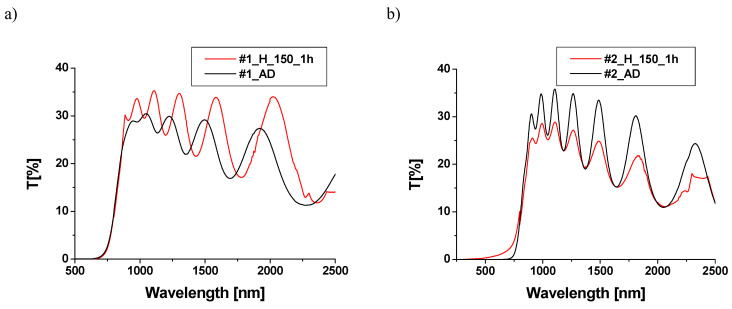

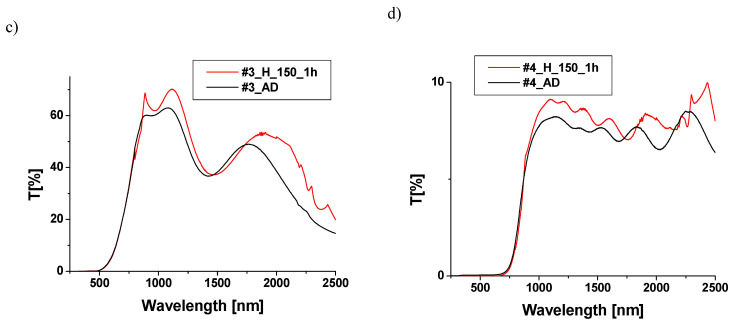
Transmission spectra of samples #1 (**a**), #2 (**b**), #3 (**c**), and #4 (**d**) before (black line) and after annealing (red line).

The spectra of transmission and reflection for TiO_2_/Cu_x_O structures were obtained with the use of the Cary 5000 spectrometer for as-deposited (_AD) structures as well as heated (_H) in air. Transmission spectra were measured in the range of 180–2500 nm. The measured transmission is the transmission of the TiO_2_/Cu_x_O structure with the glass/ITO substrate. During the transmission measurements, samples were oriented with a layer of TiO_2_ towards the incident light beam. The results are shown in Figure 5.

Samples, except for sample #3, are impermeable in the visible range. Sample #2 becomes partially permeable in this range after heating. The fundamental absorption edge of the unannealed sample #3 is shifted toward shorter wavelengths compared to other unannealed samples. Heating slightly affects the position of the fundamental absorption edge of the samples. Except for sample #4, transmission tends to decrease with increasing wavelength. The highest maximum transmission is observed in sample #3 (reaching 65% for the unannealed sample), likely due to its smaller thickness compared to other layers. Heating increases the transmission of samples #1, #3, and #4, while it decreases the transmission of sample #2. In the infrared range, the spectra of the tested samples before and after heating show numerous interference bands, indicating the homogeneity of the layer thickness [30]. These bands are not present in sample #3.

**Figure 6:**
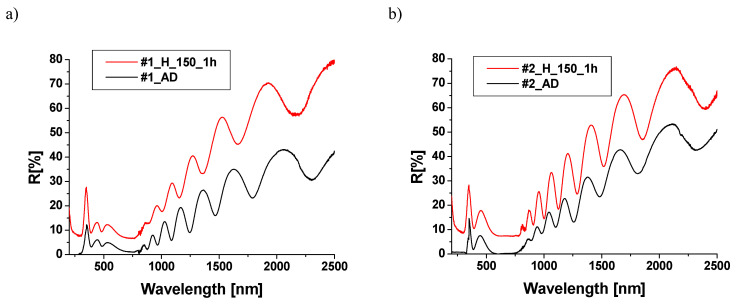

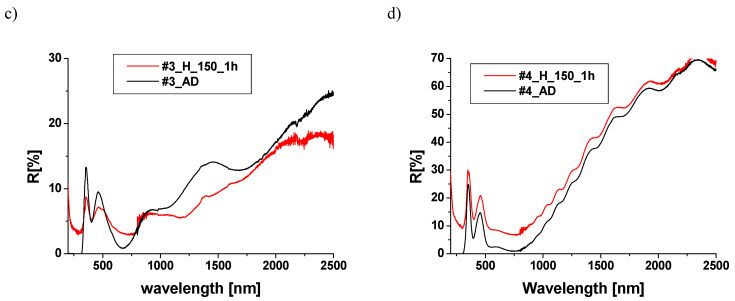
Reflection spectra of samples #1 (**a**), #2 (**b**), #3 (**c**), and #4 (**d**) from the TiO_2_ side before (black line) and after annealing (red line).

The reflection spectra of the samples were measured using the Diffuse Reflectance Accessory (DRA) system configured to measure the total reflection (i.e., Specular Component with Diffuse Component). It can be assumed that a significant portion of the reflected radiation is a specular component due to the nature of the sample surface. The reflection spectra of samples before and after heating were measured from the surface side of the TiO_2_ and Cu_x_O layers within the DRA measuring range of 180–2500 nm. The reflection spectra measured from the TiO_2_ side are shown in Figure 6.

In the ultraviolet and visible range, the reflection spectra of both sample groups—heated and unannealed—show two reflective peaks with maxima around 345 nm and 460 nm that are most likely associated with TiO_2_ in the rutile and anatase phases [31]. In the wavelength ranges of 600–800 nm and below 300 nm, the reflection for unannealed samples is negligible. Above 800 nm, both unannealed and annealed samples exhibit a monotonic increase in the reflection coefficient with increasing wavelength, with the maximum value of this reflection depending on the sample.

**Figure 7:**
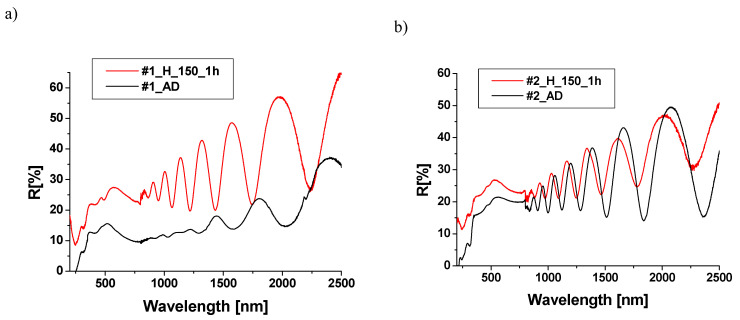

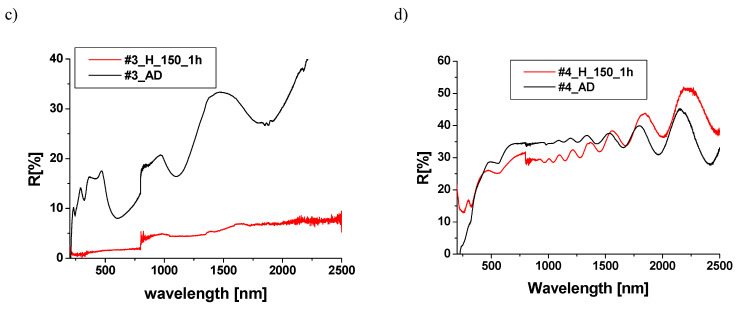
Reflection spectra of samples #1 (**a**), #2 (**b**), #3 (**c**), and #4 (**d**) from the Cu_x_O side before (black line) and after annealing (red line).

With the exception of sample #3, numerous interference bands are visible in the reflection spectra, indicating the homogeneity of the TiO_2_ layer thickness. Heating the samples results in a change in the reflection coefficient structure. For samples #1, #2, and #4, an increase in reflection is observed, while for sample #3, a decrease is noted. In the experiment, we measured total reflection, including both specular and diffuse components, with diffuse reflection being the dominant one. The increase in the roughness of the samples after annealing leads to an increase in the dominant diffusive and total reflection components. An increase in the reflection of TiO_2_/CuO structures, after annealing at temperatures ranging from 100 to 400 °C, has also been reported by other authors [24]. The reflection spectra of structures measured from the Cu_x_O layer side are shown in Figure 7.

In the reflection spectra of structures (measured from the Cu_x_O side), interference bands are visible in the infrared range, indicating the homogeneity of the thickness of the Cu_x_O layers in structures #1, #2, and #4. The reflection coefficient values in the range of 400–1000 nm (typically in the order of 10–20%) for unannealed samples #1, #2, and #3 align well with the literature values for Cu [23,32]. However, sample #4 exhibits higher reflection, reaching an average of about 35% in the infrared range.

The reflection spectrum of the unannealed sample #3 differs from the others, showing a distinct decrease in reflection, particularly around 610 nm. Heating the samples induces changes in the reflection spectra. Specifically, samples #1 and #2 exhibit an increase in the reflection coefficient compared to their pre-heating states, while samples #3 and #4 experience a decrease (particularly evident in the visible and infrared ranges). A particularly drastic decrease in the reflection coefficient was observed for sample #3, with the change in the nature of the reflection spectrum, which does not show a clear structure.

Due to the fact that TiO_2_ is transparent in a much wider area than CuO [21,33] and Cu_2_O [34], in the case of the TiO_2_/Cu_x_O, the optical properties of this structure are determined by the optical properties of CuO and Cu_2_O components, which means that the type of optical band gap can be determined by copper oxides. There is no clear statement in the literature as to whether the CuO is described by the model of direct transition [20,35] or indirect transition [19,21,36], while for Cu_2_O, the model of direct transition is adequate [34,37].

In the case of the studied structures, the model of direct transition is better suited. To determine the optical band gap energy, the Tauc model was used for direct transitions [38]:

$${\left(\alpha h\nu \right)}^{2}=A\left(h\nu -E_{g}\right),$$

where *A* is a constant, *hv* is the photon energy, *α* is the sample absorption coefficient, and d is the layer thickness which was determined on the basis of the measured reflection and transmission coefficient of structures from the following formula [39]:

$$\alpha =\frac{1}{d}ln\left[\frac{{\left(1-R\right)}^{2}}{2T}+\sqrt{\frac{{\left(1-R\right)}^{4}}{{4T}^{2}}+R^{2}}\right].$$

**Figure 8:**
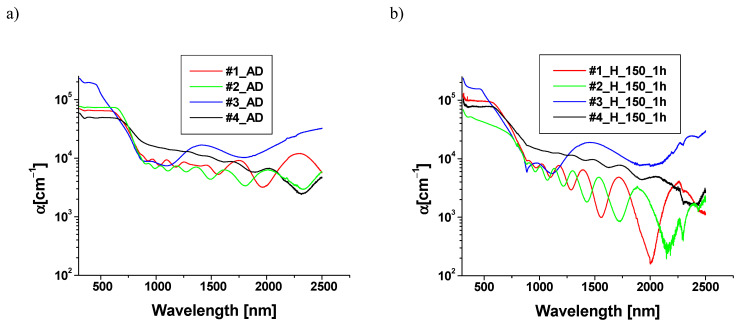
Absorption spectra of samples #1, #2, #3, and #4 before (**a**) and after annealing (**b**).

The absorption spectra of the layers before and after annealing are presented in Figure 8.

Calculated values of the optical band gap energy for the samples before and after heating are collected in Table 2.

The literature gives a wide range of optical band gap energy, varying from 1.0 eV to 4.0 eV for the thin layers of CuO [18,19,20,21,22,40,41]. It is related to the fact that the optical band gap energy of the CuO depends strongly on a number of factors, including process parameters, conditions, and methods of layer growth [20,41,42,43]. The optical band gap energy of thin Cu_2_O layers is located, in accordance with the literature [34], in a narrower range of 2.02–2.50 eV. In the case of structures #1, #3, and #4, the optical band gap energy decreases slightly after heating. The decrease in optical band gap energy after annealing in air was also observed for CuO thin films in [44]. For structure #2, we observed a significant increase in the optical band gap energy as a result of heating. This is likely due to the transition of Cu_2_O phases in sample #2 to CuO, resulting from oxidation during annealing in air at 150 °C for 1 h. Due to the absence of a Cu buffer layer between the emitter and absorber, copper does not diffuse into the structure of the Cu_x_O layer. Therefore, in the case of sample #2, the dominant process during annealing is oxidation. This transition from Cu_2_O to the CuO phase has previously been observed, for example, during selective laser heating of the Cu_2_O layer [45].

### 3.3. I-V Characterization

The I-V characteristics were measured using a Keithley 2602 I-V meter and a halogen lamp (Keithley Instruments Inc., Solon, OH, USA) as a light source with an intensity of ~800 W/m^2^ at the temperature of 40 °C, which reflects normal operating conditions (NOCTs). The obtained results present the impact of cell heating on their basic photovoltaic parameters, including I_sc_ (short-circuit current), V_oc_ (open-circuit voltage), η (efficiency), FF (fill factor), R_s_ (serial resistance), and R_sh_ (shunt resistance), which generally improved during the heating process described in Section 2. Parameters like I_sc_, V_oc_, and FF can be determined directly from light I-V characteristics, while η can be calculated from the following equation [46]:

$$\eta =\frac{FF\;I_{sc}V_{oc}}{P_{solar}}$$

where *P*_solar_ is the radiation intensity.

The series R_s_ and shunt R_sh_ resistances were calculated using the Lambert W function [47] implemented in the Origin package. The basic photovoltaic parameters of the tested as-deposited (_AD) and heated (_H) solar cells were determined from the current–voltage characteristics. Measurements were performed both in complete darkness and under illumination by a halogen lamp with a radiation intensity P_solar_ of 800 W/m^2^.

**Figure 9:**
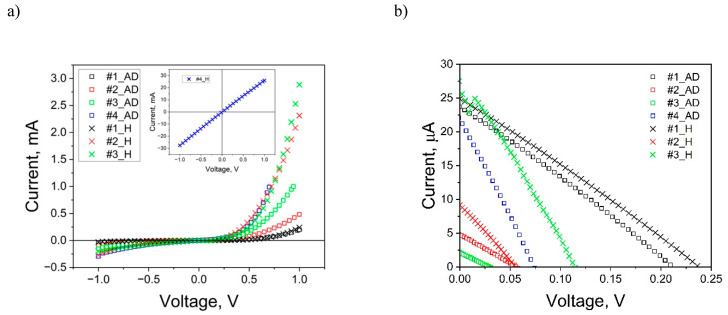
Comparison of dark (**a**) and light (**b**) I-V characteristics for heated (_H) and unheated (_AD—[27]) cell samples #1, #2, #3, and #4.

Figure 9a shows the measured dark characteristics of as-deposited and annealed cells. Characteristics for annealed cells, marked with the letter _H, show stronger non-linearity compared to the characteristics of the as-deposited structures. This suggests a lower series resistance, a more visible diode rectifying effect, and a general improvement in photovoltaic parameters after heat treatment. The exception is sample #4, for which after annealing we observe a decrease in series resistance from 253 Ω to 35 Ω and a significant decrease in shunt resistance from 4200 Ω to 1 Ω. This behavior is likely due to increased copper diffusion within the structure, leading to a short circuit. The authors had previously observed similar copper diffusion in other photovoltaic structures based on titanium oxide and copper oxide [12] as well as in CdS/CdTe thin film structures [48]. Since sample #4 no longer exhibited a photovoltaic effect after annealing, its light I-V characteristics are not shown in Figure 9b.

Figure 9b shows the light characteristics of the annealed and unheated samples, where a clear increase in the short-circuit current I_sc_ and open-circuit voltage V_oc_ is observed for all samples after annealing. An exception is sample #2, which shows a slight decrease in V_oc_ after annealing, accompanied by an increase in I_sc_. Table 3 contains the basic photovoltaic parameters for all samples exhibiting the photovoltaic effect, both before and after heating (sample #4_H does not exhibit the photovoltaic effect after annealing).

Overall, the PV parameters for samples #1_H, #2_H, and #3_H either remained stable or improved after annealing (Table 4). Notably, sample #3_H exhibited significant enhancement across all parameters but shunt resistance. Efficiency surged by eightfold, the fill factor (FF) rose from 25% to 29.1%, short-circuit current (I_sc_) escalated from 2 mA to 26 mA, and open-circuit voltage (V_oc_) soared from 29 mV to 114 mV. These enhancements are closely tied to the notable reduction in series resistance (R_s_) observed across all cells post-heating, suggesting a strong correlation between improved PV parameters and reduced R_s_. Consequently, the remarkable improvement in efficiency can be primarily attributed to this decrease in series resistance. The observed changes in photovoltaic parameters are primarily due to copper diffusion and its oxidation during annealing. The intensity of this diffusion strongly depends on the structural properties of the considered solar cells, which are significantly connected with the buffer layers being used [12,27,45,49]. For sample #1, which has a compact and homogeneous structure, diffusion is limited, and annealing does not significantly alter the I-V characteristics. For sample #2, the dominant effect during annealing is oxidation, which fills oxygen vacancies and promotes the transition from Cu_2_O to CuO. In contrast, for sample #3, for which a thin Ti buffer layer was applied, an increase in grain size and the presence of large vacancies in the crystal lattice, which depend on the configuration of the buffer layers, promote copper diffusion into the TiO_2_ layer and enhance oxidation during annealing, leading to the formation of additional CuO pathways. Furthermore, the porous structure for sample #3 promotes the filling of oxygen vacancies and the transition from Cu_2_O to CuO. As a result, sample #3 showed the most significant improvements in photovoltaic parameters after annealing. In the case of sample #4, dominant copper diffusion occurs. Limited oxidation in this sample leads to the formation of copper conduction.

## 4. Conclusions

In this paper, the authors conducted a comprehensive analysis of the effects of annealing on the optical and photovoltaic characteristics of TiO_2_/Cu_x_O cells featuring Ti and Ti/Cu buffer layers in different configurations. The study incorporated various configurations of the solar cells deposited on a substrate maintained at a constant temperature of 300 °C. Based on the optical and electrical characterization, significant changes in tested cell parameters were observed after annealing the samples. Optical characterization involved the examination of transmission and reflection spectra, revealing intriguing changes post-annealing. The transmission spectra indicated alterations in permeability and fundamental absorption edge shifts, while the reflection spectra displayed changes in reflective peaks and interference bands, suggesting structural modifications due to heating. Furthermore, the determination of the optical band gap energy elucidated the impact of annealing on the semiconductor properties of the samples. While slight decreases in band gap energy were observed for certain samples, significant variations were noted, particularly for sample #2, which were attributed to phase transitions from Cu_2_O to CuO. The I-V characterization provided the possibility to determine the photovoltaic performance, showcasing improvements in key parameters such as short-circuit current, open-circuit voltage, and efficiency post-annealing. Notably, sample #3 exhibited remarkable enhancements across all parameters, which were primarily attributed to a notable reduction in series resistance. In conclusion, this study underscores the profound influence of annealing on the optical and photovoltaic properties of TiO_2_/Cu_x_O cells, offering valuable insights into optimizing their performance for potential applications in next-generation solar cells. The parameters of TiO_2_/Cu_x_O photovoltaic structures are closely related to the intensity of copper diffusion and the processes of its oxidation as well as the elimination of oxygen vacancies. The intensity of diffusion depends, in turn, on the structural properties of each layer of the solar cell, which are significantly connected with Cu, Ti, and Ti/Cu buffer layers. Annealing of TiO_2_/Cu_x_O cells leads to intensification of diffusion and oxidation processes, which depend on the structural properties of individual layers and interfaces.

## Figures and Tables

**Table 1:** Surface roughness of TiO_2_/Cu_x_O solar cells before and after annealing analyzed by mathematic apparat Gwyddion.

Samples	Unheated (_AD)		Heated (_H)	
	S_a_, nm	S_q_, nm	S_a_, nm	S_q_, nm
#1	130.3	177.6	148.1	201.5
#2	126.1	172.7	149.1	200.6
#3	171.9	222.5	145.6	193.2
#4	135.0	185.6	133.6	186.0

**Table 2:** Optical band gap for unannealed and annealed samples #1, #2, #3, and #4.

	#1	#2	#3	#4
E_g_ [eV] before annealing	2.08	2.01	2.87	1.84
E_g_ [eV] after annealing	2.00	3.52	2.55	1.81

**Table 3:** Photovoltaic parameters for unannealed and annealed (_H) samples #1, #2, #3, and #4.

Parameter	#1 [27]	#1_H	#2 [27]	#2_H	#3 [27]	#3_H	#4 [27]	#4_H
V_oc_ [mV]	213	238	54	55	29	114	74	-
I_sc_ [mA]	24	25	4.5	9	2.0	26	22	-
η [%]	5 × 10^−3^	1 × 10^−2^	2 × 10^−4^	1 × 10^−3^	6 × 10^−5^	8 × 10^−3^	4 × 10^−3^	-
Contact area [mm^2^]	35	35	45	45	40	40	35	35
V_oc_/CuO thickness [mV/nm]	0.19	0.20	0.04	0.04	0.02	0.35	0.05	-
V_oc_/TiO_2_ thickness [mV/nm]	4.38	5.87	1.15	1.01	0.70	2.59	1.71	-
FF [%]	27	26.6	26	26.7	25	29.1	26.5	-
R_sh_ [Ω]	36,000	50,000	11,000	5900	8700	6000	4200	1
R_s_ [Ω]	968	576	1216	151	315	72	253	35

**Table 4:** Relative changes in PV parameters for annealed samples #1_H, #2_H, #3_H, and #4_H in relation to as-deposited #1, #2, #3, and #4 samples (i—improvement; d—deterioration).

Parameter	#1_H [%]	#2_H [%]	#3_H [%]	#4_H [%]
V_oc_	+12 i	+2 i	+294 i	−d
I_sc_	+4 i	+100 i	+1200 i	−d
FF	−1 d	+3 i	+16 i	−d
R_sh_	+39 i	−46 d	−31 d	−99 d
R_s_	−40 i	−88 i	−77 i	−86 i

## Data Availability

The original contributions presented in the study are included in the article, further inquiries can be directed to the corresponding author.
